# Supplementary material for: Comparison of Functional Outcomes after Anterior Cruciate Ligament Reconstruction with Meniscal Repair for Unstable versus Stable Meniscal Tears
Source: Diagnostics (Basel). 2024 Apr 23;14(9):871. doi: 10.3390/diagnostics14090871 (PMC11083838; doi:10.3390/diagnostics14090871)
Supplement: Supplementary file 1 [file diagnostics-14-00871-s001.zip › diagnostics-2926161-supplementary.pdf]

Rehabilitation Protocol

Phase 1: Immediately Postoperatively to Week 6

Condition	Stable	Unstable
Precautions	-PWB 0-2 weeks<br>-Tolerable WB at 2-6 weeks<br>-ROM 0°~90° at 2 weeks<br>-Isometric OKC at 3 weeks~	-NWB<br>-ROM 0°~90° at 4 weeks<br>-Isometric OKC at 3~7 week<br>-No OKC hamstring exercise
Goals/Criteria to Advance	-Pain control<br>-Reducing swelling<br>-Recovering ROM<br>-Normalizing gait<br>-ROM symmetrical<br>-Normal joint temperature<br>-Minimal to no joint effusion<br>*Unstable: adjusted ROM and WB timelines	
Exercises	-Patella mobilization<br>-Quad sets<br>-Self ROM (wall slides, heel slides, heel prop extension overpressure)<br>-Ankle pumps<br>-Straight leg raises with NMES<br>-Terminal knee extension (OKC, consider NMES)<br>-Calf/hamstring stretching<br>-4-direction (flexion, extension, adduction, abduction) leg strengthening in full extension with elastic band<br>-Sitting knee extension (isometric -> band exercise, starting at 4 weeks)<br>*Unstable: adapted for NWB and delayed timelines	

Abbreviation: PWB, partial weight bearing; NWB, non-weight bearing; ROM, range of motion; OKC, open kinetic chain; NMES, neuromuscular electrical stimulation.

Phase 2: Postoperatively Weeks 6 to 12

Condition	Stable	Unstable
Precautions	-FWB at 6 weeks<br>-Full ROM at 6 weeks<br>-CKC 90° at 10 weeks	-Tolerable WB at 6-8 weeks<br>-FWB at 8 weeks<br>-Full ROM at 8 weeks<br>-OKC at 6 weeks (hamstring at 10 weeks)<br>-CKC 60° at 8~16 weeks<br>-Single-leg balance at 8 weeks
Goals/Criteria to Advance	-Muscle strengthening & proprioception training<br>-Achieve full WB<br>-Normalize gait pattern on flat ground<br>-Maintain trace to no joint effusion	
Exercises	-Progressive ROM<br>-PWB to FWB gait training with and without crutches and brace<br>-Calf/Hamstring stretching and strengthening<br>-Weight shifting and bearing (single-leg balance)<br>-Isometric wall squat (limited ROM)<br>-Leg press (limited ROM, tolerable)<br>-Progressive balance training (stable to unstable surface/both to single)<br>-Hip strengthening (clam shell, hip bridge, hip abduction and etc.)<br>-Core training (planks, dead bugs and etc.)<br>-Stationary biking, CKC training (squat, lunge, leg press, step ups/downs, bridges and etc.)<br>-OKC training (leg extension/curl, with elastic band to machine leg extension/curl)	

Abbreviation: FWB, full weight bearing; PWB, partial weight bearing; WB, weight bearing; ROM, range of motion; OKC, open kinetic chain; CKC, closed kinetic chain; NMES, neuromuscular electrical stimulation
**Exercise parameters: 3sets, 15 to 25 repetitions, 30 to 60 second rest periods, 3 to 4 times per week*

Phase 3: Postoperatively Weeks 13 to 24

Condition	Stable	Unstable
Precautions	-Running at 16 weeks	-CKC 90° at 16 weeks<br>-Running at 20 weeks
Goals/Criteria to Advance	-Enhance muscle strength<br>-Proprioception, and neuromuscular control (plyometric and agility)<br>-Achieve 90% LSI scores (isokinetic test)<br>-Symmetric balance scores<br>-85% Single-leg hop scores	
Exercises	-Transition Gym-based program and/or supervised training<br>-Cardiovascular training (biking and running)<br>-Advance motor control training<br>-High intensity and plyometric training<br>-Progressive motor control training<br>-Progressive running training (distance)<br>-Agility training (side steps, ladder drills, one or double jump and etc.)	

Abbreviation: CKC, closed kinetic chain; LSI, limb symmetry index.

**Exercise parameters: 3sets, 8 to 12 repetitions, 2-to-3-minute rest periods, 3 times per week*

Phase 4: Postoperatively Weeks 24+

Condition	Stable	Unstable
Precautions	-Specific RTS training at 24 weeks	-Specific RTS training at 36 weeks
Goals/Criteria to Advance	-Sport-specific technical training<br>-90% LSI scores<br>-Symmetric balance scores<br>-90% Single-leg hop scores	
Exercises	-High intensity power and maximal effort strength training<br>-Progressive agilities and motor control training<br>-Specific training for individualized to sport/position<br>-Regular evaluations of strength, postural stability, and functional performance (at 9 and 12 months)<br>-Education for secondary prevention	

Abbreviation: RTS, return to sports; LSI, limb symmetry index.
**Single-leg hop: Single hop distance, Side hop*
